# Assisted Reproductive Technology and Obstetric Outcome in Couples when The Male Partner Has A Chronic Viral Disease

**Published:** 2013-12-22

**Authors:** Irene Molina, María Carmen del Gonzalvo, Ana Clavero, Miguel Ángel López-Ruz, Juan Mozas, Juan Pasquau, Antonio Sampedro, Luis Martínez, José Antonio Castilla

**Affiliations:** 1 Human Reproduction Unit, Clinical Management Unit for Obstetrics and Gynaecology, Virgen de las Nieves University Hospital, Granada Institute for Healthcare Research, Granada, Spain; 2 Infectious Disease Unit, Internal Medicine Service, Virgen de las Nieves University Hospital, Granada, Spain; 3 Biomedical Research Centre Network for Epidemiology and Public Health (CIBERESP), University of Granada, Spain; 4 Microbiology Service, Virgen de las Nieves University Hospital, Granada, Spain

**Keywords:** HIV, HCV, HBV, Reproductive Techniques, Obstetric Labor Complications

## Abstract

**Background:**

Assisted reproductive technology (ART) with washed semen can achieve pregnancy with minimal risk of horizontal and vertical transmission of chronic viral diseases (CVD) such as human immunodeficiency virus (HIV), hepati- tis C virus (HCV) and hepatitis B virus (HBV) among serodiscordant couples. How- ever, few studies have been made of the use made by these couples of ARTs or of the obstetric results achieved.

**Materials and Methods:**

In this retrospective study, 93 men who were seropositive for HIV, HCV or HBV and who underwent assisted reproduction treatment at our centre (Hospital Universitario Virgen de las Nieves, Granada, Spain) were included. Washed semen was tested to detect viral particles. Non-infected women were tested before and after each treatment, as were the neonates at birth and after three months.

**Results:**

A total of 62 sperm samples were washed, and none were positive for the detec- tion of viral molecules. Semen samples from 34 HBV positive males were not washed since the female partner had immunity to hepatitis B. In total, 38 clinical pregnancies were achieved (22% per cycle and 40.9% per couple) out of 173 cycles initiated, and 28 births were achieved (16.2% per cycle and 30.1% per couple), producing 34 live births. The rate of multiple pregnancies was 21.4%. Obstetric and neonatal results were similar in the groups of couples studied. At follow-up, no seroconversion was detected in the women or neonates.

**Conclusion:**

Sperm washing and intracytoplasmic sperm injection are shown to be a safe and effective option for reducing the risk of transmission or super infection in serodiscordant or concordant couples who wish to have a child. Pregnancies ob- tained by ART in couples when the male is CVD infected achieve good obstetric and neonatal results.

## Introduction

Assisted reproductive technology (ART) first came into use to address problems of infertility, and was subsequently applied to fertile couples with the aim of reducing the risk of transmission of genetic and infectious diseases. With this latter objective, ART has been applied to couples in which one or both partners are infected by human immunodeficiency virus (HIV), hepatitis C virus (HCV) or hepatitis B virus (HBV). Semprini et al. (1) recorded the first birth achieved after using washed semen from an HIV seropositive man. Today, the reproduction options for serodiscordant couples with a chronic viral disease (CVD) have been expanded. Among these possibilities are unprotected intercourse and timed intercourse with or without pre-exposure prophylaxis (PREP), ART using semen washed with or without testing for detectable viral load, donor sperm, the donation of embryos from seronegative couples, and adoption. However, many of these options are not accepted by the couple (2) or are not recommended by physicians, and so the option of washed semen is most often adopted (3).

To date, many studies have described the safety of the semen wash-intracytoplasmic sperm injection (ICSI) technique for couples that are serodiscordant for HIV (4). However, only a few of these studies (5- 8), including the largest series to date, of over 3000 treatment cycles, published by CREAThE (9), have reported obstetric and neonatal results for the correct evaluation of ART results, as has been recommended by different groups (10).

A similar situation occurs with studies analysing the use of ART for couples in which the male partner is seropositive for HCV or HBV (11-18). In such cases, obstetric results are much more limited.

Since late 2005, the Human Reproduction Unit at Hospital Universitario Virgen de las Nieves, Granada, Spain has been the only one in the public health system of Andalucia (8.2 million inhabitants) providing fertility care for couples with an HIV, HCV or HBV infection. We follow the recommendations of the ethics taskforce of the european society of human reproduction and embryology (ESHRE) about suitable treatment compliance, avoidance of other risk factors such as drug abuse, treatment in reference centres with established protocols, a separate adapted laboratory, as well as separate tanks for storage of infected material, and appropriate multidisciplinary support (19).

The aim of this retrospective study was to determine, for couples in which the male was seropositive for HIV, HCV or HBV: i. the efficiency of sperm wash in terms of viral load; ii. the results of ART-ICSI; iii. the seroconversion rates after the treatment; and iv. the obstetric and neonatal outcome for such couples at a public hospital.

## Materials and Methods

A retrospective review was conducted of men who were seropositive for HIV, HCV or HBV and underwent assisted reproduction treatment between November 2005 and December 2009.

To be enrolled, all the couples were required to sign informed consent, and to attest to safe sex practices since four months before beginning the treatment and not to have sex from one month before until one month after the end of the treatment. Male and seropositive female partners were requested to be under the care of an infectious disease specialist and to provide a full report of their disease including current serological study, CD4 counts in blood (only for HIVseropositive), blood viral load with a maximum age of 4 months and treatment received and evolution of the disease for the past 12 months. To enrol an HIV positive female, both undetectable viral load and CD4 counts >200 cells/mm3 were required. Seronegative female partners were required to provide a current serological study and a blood viral load. HBV negative females with HBV positive male partners were vaccinated, and if immunity was not achieved, the partner’s semen was washed and viral load was determined. The couples had to wait an average of two years for ICSI treatment, as our hospital is a public one and it has a waiting list. Every couple was allowed a maximum of two attempts (two cycles with embryo transfer) to achieve a pregnancy.

A standard evaluation was performed on both partners, consisting of anamnesis and physical examination. Female fertility was also assessed by gynaecological examination, a vaginal ultrasound examination of the uterus and ovaries, a vaginal sample for bacteriological testing and a smear test. Basal hormonal study was determined from blood samples on day 3 of the menstrual cycle. The men were also subjected to seminal analysis, following world health organization (WHO) criteria (20). The ART laboratory used for all procedures was separated from the laboratory facilities used for couples negative for HIV, HBV and HCV.

Semen samples were obtained after sexual abstinence of 3 days, and subsequently kept and manipulated in a class II biological safety cabinet. After liquefaction, semen parameters were evaluated as outlined by the WHO (20) and samples were processed by centrifuging (20 minutes at 300 g) through a discontinuous density gradient 80-40% (PureSperm 100 and PureSperm Buffer, Nidacon International AB, Mölndal, Sweden) with 1 ml per layer, with the semen pippetted directly on top of the upper layer. Pellets were washed 1:2 (vol: vol) with PureSperm Wash (Nidacon, Mölndal, Sweden) for 8 minutes at 300 g, then the supernatant was removed and the pellet was resuspended in 3 ml of Pure Sperm Wash. The concentration was assessed and the sample was divided in two, and one half was then used to test for viral presence. The other half was frozen with Sperm Freezing Medium (Irvine Scientific, Santa Ana, CA), and stored until needed for use, after the PCR test for HIV resulted negative.

Quantification of HIV RNA, HCV RNA and HBV DNA in final processed semen was performed by real-time PCR COBAS TaqMan™ 96 instrument (COBAS Ampliprep™ analyser; Roche Diagnostics GmbH, Manheim, Germany).

Ovarian stimulation protocols were selected according to clinical data, patient’s age and hormonal profile and the result of any previous stimulation. Normally, the long protocol was adopted for the first cycle, and then, for the second one, the long or the short protocol was chosen depending on the results of the first cycle. The cycles were monitored by serial transvaginal ultrasound examination and serum estradiol (E2) levels. When the follicles had reached a mean diameter of 18-22 mm, human chorionic gonadotropin (hCG) was administered, and 34-36 hours later, oocyte retrieval was performed with ultrasound guidance. The oocytes collected were incubated in GIVF Plus supplemented with human serum albumin (HSA, Vitrolife, Göteborg, Sweden) and the mature oocytes with extrusion of the first polar body were then microinjected with sperm selected from the motile fraction. The procedure was performed as previously described (21). The embryos were incubated in G-1 Plus supplemented with HSA (Vitrolife, Göteborg, Sweden) in incubators separated from those of seronegative infertile couples and transferred at day 2-3 into Embryoglue (Vitrolife, Göteborg, Sweden). Progesterone (400 mg/day) and folic acid (0.4 mg/ day) were prescribed on the day of oocyte retrieval and maintained until the patient was instructed to suspend this medication. The remaining embryos were vitrified for possible future transfers.

Complications such as ovarian hyperstimulation syndrome (OHSS) were noted. Clinical pregnancy was assessed by ultrasonographic visualization of one or more gestational sacs or definitive clinical signs of pregnancy as described by the International Committee for Monitoring Assisted Reproductive Technology. Delivery was defined as the expulsion or extraction of one or more foetuses from the mother after 20 completed weeks of gestational age, and live birth was considered to be the complete expulsion or extraction from its mother of a product of fertilization, irrespective of the duration of the pregnancy, which, after such separation, breathes or shows any other evidence of life such as heart beat, umbilical cord pulsation, or definite movement of voluntary muscles, irrespective of whether the umbilical cord has been cut or the placenta remains attached (22).

Comparisons of rates between groups were performed using chi-squared tests. All the tests were twosided, with a p value of 5% considered as significant.

The blood viral load of the female partner was tested using RNA polymerase chain reaction (PCR) at 3 weeks, 3 months and 6 months after the ART. If the woman became pregnant, the blood viral load was tested every 2 months, and the serological study performed every 3 months. The infants were also tested at birth and at age 3 months.

## Results

After considering a total of 105 couples, 93 couples were included in this study and twelve were rejected as they did not meet the inclusion criteria; two men had active drug consumption, three men did not present adequate adherence to highly active antiretroviral therapy (HAART), another two men had active opportunist infection and five women were aged older than 40 years.

In 33 of the couples treated, the male was HIV seropositive (23 men were also HCV positive, one was HBV positive and another was both HCV and HBV positive). In another 23 couples, the male was HCV seropositive (one man was also HBV positive), and in the remaining 37 couples, the male was HBV seropositive. The men were infected with different genotypes of HCV: 43.5% (20 men) with genotype 1, 6.5% (n=3) with genotype 2, 26.1% (n=12) with genotype 3 and 23.9% (n=11) with genotype 4. The characteristic profile of the couples who underwent ICSI is shown in table 1.

**Table 1:** Characteristic profile of couples undergoing IVF-ICSI


Reference	HIV n = 33		HCV n= 23		HBV n= 37	
	mean±SD	Range	mean±SD	Range	mean±SD	Range

**Female partner**						
**Age (Y)**	34.6±4.1	25-40	34.2±2.8	29-39	34.0±3.8	26-40
**HIV, n (%)**	2(6.1)					
**Hepatitis C, n (%)**	1(3)		1(4.4)			
**Hepatitis B, n (%)**	0(0)		0(0)			
**Known HIV/HVC/HVB diagnosis (Y)**	6.5±2.1	5-8	5±0			
**On HAART therapy, n (%)**	2(100)					
**HVC treatment, n (%)**	0(0)		0(0)			
**Viral load (ui/mL)**			376000			
**Undetectable viral load HIV, n (%)**	2(100)					
**Undetectable viral load HVC, n (%)**	0(0)		0(0)			
**Undetectable viral load HVB, n (%)**	0					
**CD^4^^+^ T-cell count (mm^3^)**	666±5.7	662-670				
**HVB immunity, n (%)**					34(91.9)	
**Male partner**						
**Age (Y)**	40.1±5.7	28-55	39.4±6.1	32-50	36.4±3.8	26-44
**Hepatitis C, n (%)**						
**Hepatitis B, n (%)**	2(6.1)		1(4.4)			
**Known HIV/HVC/HVB diagnosis (Y)**	11±4.8	2-19	17±6.1	10-21	10.3±8.5	4-20
**On HAART therapy, n (%)**	28(84.9)					
**HVC treatment, n (%)**	3(23.1)		3(23.1)			
**Viral load (ui/mL)**	31631.5±47110.4*	68-123000	3367025.6±4075433.2	1071-15937448	1837.5±2577.3	17-10481.1
**Undetectable viral load HIV, n (%)**	27(81.8)					
**Undetectable viral load HVC, n (%)**	7(30.4)		3(13)			
**Undetectable viral load HVB, n (%)**	1(50)		1(100)		7(18.9)	
**CD^4^^+^ T-cell count (mm^3^)**	569.3±296.6	109-1654				


* ; Viral load (VIH??HIV) in copies/ml

A total of 62 sperm washes from 59 couples were performed, and none were positive for the detection of viral molecules. The semen samples from the 34 HBV-positive males were not washed since the female partner had immunity to hepatitis B.

The results of our ICSI programme, with respect to viral infection, are summarized in table 2. A total of 173 cycles were performed for 93 couples, whereas 25 cycles (14.5%) were cancelled before oocyte retrieval: 2 cycles (8%) due to the presence of an ovarian cyst, 19 cycles (76%) because of low response, 2 cycles (8%) due to hyper-response of the woman, one cycle (4%) due to failure of down-regulation, and one couple failed to attend for oocyte retrieval.

**Table 2:** IVF performance and outcome of IVF-ICSI cycles


	HIV(n=33)		HIV(n=23)		HIV(n=37)	
	Mean ± SD	Range	Mean ± SD	Range	Mean ± SD	Range

**Number of cycles initiated, n**	61		48		64	
**Total FSH**	2409.6 ± 1153.8	1013-5550	2305 ± 964.3	1275-4725	2315.5 ± 845.8	825-4875
**Days of stimulation**	11.2 ± 2.7	7-22	10.7 ± 2.2	7-16	10.6 ± 1.9	8-18
**Follicles >17 mm**	5.3 ± 4.1	0-15	5.7 ± 3.4	0-13	5.6 ± 3.1	1-14
**Peak estradiol level (pg/ml)**	2760.6 ± 1408.2	672-6816	2536.4 ± 1417.1	759.8-6230	2214.6 ± 1302.6	263-5240
**LH (peak estradiol)**	2.4 ± 1.7	0.30-8	2.8 ± 1.9	0.21-9	2.4 ± 1.6	0.1-6.1
**Coasting, n (%)**	5 (8.2)		1 (2.1)			
**Days of coasting**	1.8 ± 1.3	1-4	5 ± 0		0 ± 0	
**Number of retrievals**	48		43		56	
**Cycle cancellation rate, n (%)**	13 (21.3)		4 (8.3)		8(12.5)	
**Number ofoocytes retrieved per retrieval**	10.6 ± 6.1	1-23	9.4 ± 4.6	0-20	9.5 ± 5.2	0-19
**Number of mature oocytes for ICSI per retrieval**	8.5 ± 5.4	1-20	7.6 ± 4.1	0-15	7.5 ± 4.4	1-18
**Number of normally fertilized oocytes per retrieval**	4.6 ± 3.6	0-17	4.1 ± 2.9	0-12	4.5 ± 3.4	0-15
**Rate of normally fertilized oocytes per retrieval, (%)**	54.6 ± 29.1	0-100	52.3 ± 21.1	0-100	60.5 ± 28.4	0-100
**Embryo transfers**						
**Retrievals with not enough embryos for fresh transfer, n (%)**	6 (18.2)		5 (11.6)		3 (5.4)	
**Number of embryos transferred per embryo transfer**	1.9 ± 0.4	1-3	2.1 ± 0.5	1-3	2.0 ± 0.5	1-3
**Implantation rate fresh (%)**	13.2		16.9		16.8	
**Implantation rate thaw (%)**	22.2		0		45.5	
**Cycles with cryopreservation**						
**Retrievals with enough embryos for fresh transfer and cryopreservation, n (%)**	7 (14.6)		5 (11.6)		12 (21.4)	
**Retrievals with cryopreservation (no fresh transfer), n (%)**	2 (4.2)		0 (0)		0 (0)	
**Number of embryos cryopreserved per cryopreservation**	1.9 ± 0.4	1-3	1.4 ± 0.9	1-3	2.8 ± 2	1-8
**Number of embryos thawed, n (%)**	19 (59.4)		5 (71.4)		16 (48.5)	
**Number of embryos survived and transferred after thawing (cryosurvival), n (%)**	9 (47.4)		2 (0)		11 (68.8)	
**OHSS, n (%)**	1 (1.6)		0 (0)		0 (0)	


With respect to the different infection diseases, there were no significant differences in the number of oocytes retrieved, the number of mature oocytes, the fertilization rate, the number of embryos transferred or the number of embryos cryopreserved per retrieval. In the case of two women, no transfer was performed, due to ovarian hyper stimulation syndrome (OHSS) and cystitis.

The mean fertilization rate achieved was 56.2%, with a mean implantation rate of 15.7% for fresh transfers and 31.8% for thawed embryos. In total, 38 clinical pregnancies (22% per cycle and 40.9% per couple) took place, with 28 live births delivered (16.2% per cycle and 30.1% per couple). The miscarriage rate was 26.3% and that of multiple pregnancies, 21.4% (Table 3). As a result, 34 newborns ( 22 singles and 6 twins) were delivered (Table 4). Preterm deliveries (<37 weeks) occurred in 8 neonates, mostly arising from multiple gestation. Extreme prematurity (<32 weeks) was reported in one neonate, 6 babies were born with low birth weight (<2500 g) and one with very low birth weight (<1500 g). No significant differences were recorded in fertilization, pregnancy rates, obstetric or neonatal results for the different groups of CVD.

No seroconversion was detected in any of the 34 newborns (tested at birth and at age 3 months) or in the 62 women treated with washed sperm during assisted reproduction programmes.

**Table 3:** Pregnancy data


	HIV		HCV		HBV	
	n	(%)	n	(%)	n	(%)

**Intrauterine death**	0		0		0	
**Clinical pregnancy rate fresh**	8		10		14	
**Per IVF cycle**		(13.1)		(20.8)		(21.9)
**Per retrieval **		(16.7)		(23.3)		(25)
**Per embryo transfer fresh**		(20)		(27.0)		(27.5)
**Per couple**		(24.2)		(43.5)		(37.8)
**Clinical pregnancy rate thaw**	2		0		4	
**Per thaw cycle**		(33.3)		(0)		(44.4)
**Per embryo transfer thaw**		(40)		(0)		(66.7)
**Clinical pregnancy rate total**	10		10		18	
**Per IVF cycle**		(16.4)		(20.8)		(28.1)
**Per retrieval**		(20.8)		(23.3)		(32.1)
**Per embryo transfer thaw**		(22.2)		(25.6)		(31.6)
**Per couple**		(30.3)		(43.5)		(48.6)
**Miscarriage rate **	2	(20)	2	(20)	6	(33.3)
**Ectopic**	0	(0)	0	(0)	0	(0)
**Live birth delivery rate fresh**	7		8		9	
**Per IVF cycle**		(11.5)		(16.7)		(14.1)
**Per retrieval**		(14.6)		(18.6)		(16.1)
**Per embryo transfer fresh**		(17.5)		(21.6)		(17.6)
**Per couple**		(21.2)		(34.8)		(24.3)
**Live birth delivery rate thaw**	1		0		3	
**Per thaw cycle**		(16.7)		(0)		(33.3)
**Per embryo transfer thaw **		(20)		(0)		(50)
**Live birth delivery rate total**	8		8		12	
**Per IVF cycle **		(13.1)		(16.7)		(18.8)
**Per retrieval**		(16.7)		(18.6)		(21.4)
**Per embryo transfer**		(17.8)		(20.5)		(21.1)
**Per couple**		(24.2)		(34.8)		(32.4)


**Table 4:** aa


	HIV		HCV		HBV	
	mean±SD	Range	mean±SD	Range	mean±SD	Range

**Total number couples delivered**	8		8		12	
**Total number of infants delivered**	10		10		14	
**Total number of deliveries**						
**Singletons, n (%)**	6(75)		6(75)		10(83.3)	
**Twins, n (%)**	2(25)		2(25)		2(16.7)	
**Triplets, n (%)**	0(0)		0(0)		0(0)	
**Multiple gestation rate, n (%)**	2(25)		2(25)		2(16.7)	
**Vaginal birth, n (%) **	6(75)		3(37.5)		9(75)	
**Cesarean section, n (%)**	2(25)		5(62.5)		3(25)	
**Full- Term delivery (≥ 37 weeks), n**	6		7		9	
**Nº infants, n (%)**	8(80)		8(80)		9(64.3)	
**Gestational age (weeks)**	39±1.5	38-42	39±1.5	37-41	38±1	37-40
**Birth weight (grams)**	2925.6±2630	2630-3315	2809±470.2	2150-3700	3376±353.7	2760-3890
**Preterm delivery (<37 weeks gestation), n **	1		1		3	
**Number of infants, n (%)**	1(10)		2(20)		5	
**Gestational age (weeks)**	36±0	36-36	34±0		36±0.6	35-36
**Birth weight (grams)**	2880±0	2880-2880	2342.5±343	2100-2585	2286±531.6	1840-3200
**Foetal death, n (%)**	0(0)		0(0)		0(0)	
**Maternal seroconversion, n (%)**	0(0)		0(0)		0(0)	
**Delivered offspring seroconversion, n (%)**	0(0)		0(0)		0(0)	
**Extreme prematurity (<32 weeks), n**	1		0		0	
**Nº infants, n (%)**	1(10)		0(0)		00	
**Low birth weight (<2500 grams), n**						
**Nº infants, n (%)**	0(0)		2(20)		4(28.6)	
**Birthweight**	-		2125±35.4	2100-2150	2057.5±169.2	1840-2215
**Very low birth weight (<1500 grams) ,n**	1		0			
**Nº infants, n (%)**	1(10)		0(0)		0(0)	
** Birth weight**	750±0					


## Discussion

Our data show that sperm wash testing for detectable viral load of final processed semen and ICSI seems to be a safe and effective option for serodiscordant couples to conceive and to avoid transmitting the virus to the mother and child.

It is currently estimated that following recent advances in antiretroviral treatment, when total suppression of the viral load is achieved, the risk of sexual transmission is 1:100000 (23). When ICSI is combined with routine viral detection testing of the final processed semen before using the sample, the risk of transmission is significantly reduced (24-26). In the case of HCV, sexual transmission had been considered to occur only rarely (27, 28). However, recent data indicate that sexual transmission of HCV can occur, especially among HIV-infected persons. Centers for Disease Control and Prevention surveillance data show that 10% of persons with acute HCV infection report contact with a known HCV-infected sex partner as their only risk of infection (29). For the case of HBV, it has been reported that around 25% of stable sexual contacts between patients with chronic HBV infection experience seroconversion (30), although vaccination against HBV drastically reduces the risk of sexually transmitted infection (31).

To date, no seroconversions have been reported following ICSI treatment in which semen samples are processed by density gradient centrifugation of sperm, with or without routine testing of final semen processes (4). In our study, no seroconversions were observed, either among the 34 neonates (tested at birth and at age 3 months), or among the 62 women treated with washed sperm during assisted reproduction programmes.

Some assisted reproduction centres perform ICSI for all couples, rather than Intrauterine insemination (IUI), justifying this on the greater success rate, and therefore, less exposure to possibly contaminated sperm, or on the theory that ICSI requires only in vitro contact between a single sperm and egg, which should dramatically reduce the risk of transferring the viral particles that are often present in the semen or seminal cellular compartment (5, 32).

Other authors consider this theory to be unproven, and believe the process could involve the introduction of viral particles directly into the oocyte, with as yet unknown effects (33-35), as well as imposing higher costs and greater demands on healthcare staff, and being associated with a higher number of obstetric complications. At our hospital, ICSI is routinely applied to all couples, because as is the case at other centres (18, 36), and we do not also possess analytical results with which to discount the presence of viral particles on the same day as the sperm washing is performed. Therefore, the semen must be frozen, and in consequence, we do not obtain sufficient motile sperm for IUI to be performed.

Considerable differences have been reported in the percentage of positive results for RNA-HIV of final processed semen, ranging from 0% (37) to 40% (38). Similar discrepancies have been reported for HCV, with detection rates ranging from 0 (39, 40) to 57% (41). These differences may be due to the heterogeneity of the patients treated, to the sensitivity of the PCR kits used or to the elimination of PCR inhibitors (42). For this reason, it is essential that all concerned, like ourselves and other researchers, should participate in external quality control programmes (43, 44).

Our results for clinical and ongoing pregnancies achieved for couples in which the male partner is HIV positive are similar to those described by other authors (18, 45), although lower than those reported by some (7, 8). This could be because the mean number of embryos transferred by the latter (3.54 and 3, respectively) is significantly higher than our value of 2 embryos. However, our multiple pregnancy rates (21.4%) is lower than that obtained by these authors (57.1 and 41%, respectively), and is similar to that obtained in another study, in which fewer embryos were transferred (46). It should be taken into account that many of these patients are fertile couples, with no reproductive problems, and therefore, the number of embryos transferred should be minimised in order to reduce the risk of multiple pregnancy.

With respect to clinical and ongoing pregnancies for couples with HCV and HBV-positive male partners, our results are similar to those published by other authors (13, 15, 18).

Although we found no statistically significant differences in the pregnancy rates among the three types of couples studied, the trend for rates to be lower among the HIV-infected couples could be related to reduce fertility rate among HIV-infected men as a result of treatment or infection (47, 48). With respect to the difference in implantation rates observed among all groups, between fresh and thawed embryos, this could be due to different endometrial receptivity and greater synchronisation between the embryo and the endometrium in cycles with thawed embryos (49). In turn, this factor could result from the fact that endometrial development in frozen-thawed cycles can be controlled more precisely than in cycles of controlled ovarian hyperstimulation with gonadotropins (50).

Although not expressly described, we found no significant differences between embryology laboratory results for males who were only HIV seropositive, and those who were positive for both HIV and HCV. This has also been reported by other authors, in studies of these two groups of patients, or comparing them with the results obtained from healthy couples (14, 51).

Although the rate of caesarean delivery was higher among the group in which the male partner was HCV infected, these differences were not statistically significant. Moreover, the overall rate of caesareans in our study (35.7%) was lower than that described by other authors for this type of couple (6-8). The obstetric and neonatal complications recorded were similar to those published in the literature for non-serodiscordant couples (52, 53).

## Conclusion

We showed sperm washing and ICSI is a safe and effective option for reducing the risk of transmission or super infection in serodiscordant or concordant couples who wish to have a child. The pregnancies obtained by ART among couples in which the male partner has CVD produce good obstetric and neonatal results.

## References

[1] Semprini AE, Levi-Setti P, Bozzo M, Ravizza M, Taglioretti A, Sulpizio P (1992). Insemination of HIV-negative women with processed semen of HIV-positive partners. Lancet.

[2] Klein J, Pena JE, Thornton MH, Sauer MV (2003). Understanding the motivations, concerns, and desires of human immunodeficiency virus 1-serodiscordant couples wishing to have children through assisted reproduction. Obstet Gynecol.

[3] Ethics Committee of the American Society for Reproductive Medicine (2010). Human immunodeficiency virus and infertility treatment. Fertil Steril.

[4] Eke AC, Oragwu C (2011). Sperm washing to prevent HIV transmission from HIV-infected men but allowing conception in serodiscordant couples. Cochrane Database Syst Rev.

[5] Sauer MV, Chang PL (2002). Establishing a clinical program for human immunodeficiency virus 1-seropositive men to father seronegative children by means of in vitro fertilization with intracytoplasmic sperm injection. Am J Obstet Gynecol.

[6] Cleary-Goldman J, Pena JE, Thornton MH 2nd, Robinson JN, D’Alton ME, Sauer MV (2003). Obstetric outcomes of human immunodeficiency virus-1-serodiscordant couples following in vitro fertilization with intracytoplasmic sperm injection. Am J Perinatol.

[7] Peña JE, Thornton MH, Sauer MV (2003). Assessing the clinical utility of in vitro fertilization with intracytoplasmic sperm injection in human immunodeficiency virus type 1 serodiscordant couples: report of 113 consecutive cycles. Fertil Steril.

[8] Sauer MV, Wang JG, Douglas NC, Nakhuda GS, Vardhana P, Jovanovic V (2009). Providing fertility care to men seropositive for human immunodeficiency virus: reviewing 10 years of experience and 420 consecutive cycles of in vitro fertilization and intracytoplasmic sperm injection. Fertil Steril.

[9] Bujan L, Hollander L, Coudert M, Gilling-Smith C, Vucetich A, Guibert J (2007). Safety and efficacy of sperm washing in HIV- 1-serodiscordant couples where the male is infected: results from the European CREAThE network. AIDS.

[10] ESHRE Capri Workshop Group (2007). Intracytoplasmic sperm injection (ICSI) in 2006: evidence and evolution. Hum Reprod Update.

[11] Garrido N, Meseguer M, Bellver J, Remohi J, Simon C, Pellicer A (2004). Report of the results of a 2 year programme of sperm wash and ICSI treatment for human immunodeficiency virus and hepatitis C virus serodiscordant couples. Hum Reprod.

[12] Pirwany IR, Phillips S, Kelly S, Buckett W, Tan SL (2004). Reproductive performance of couples discordant for hepatitis B and C following IVF treatment. J Assist Reprod Genet.

[13] Mencaglia L, Falcone P, Lentini GM, Consigli S, Pisoni M, Lofiego V (2005). ICSI for treatment of human immunodeficiency virus and hepatitis C virus-serodiscordant couples with infected male partner. Hum Reprod.

[14] Chu MC, Pena JE, Nakhuda GS, Thornton MH 2nd, Sauer MV (2006). Assessing the reproductive performance of men co-infected with HIV-1 and hepatitis C undergoing assisted reproduction. Arch Gynecol Obstet.

[15] Ohl J, Partisani M (2007). The desire to become a parent when infected with human immunodeficiency virus, hepatitis C virus or hepatitis B virus. Gynecol Obstet Fertil.

[16] Bourlet T, Lornage J, Maertens A, Garret AS, Saoudin H, Tardy JC (2009). Prospective evaluation of the threat related to the use of seminal fractions from hepatitis C virus-infected men in assisted reproductive techniques. Hum Reprod.

[17] Lam PM, Suen SH, Lao TT, Cheung LP, Leung TY, Haines C (2010). Hepatitis B infection and outcomes of in vitro fertilization and embryo transfer treatment. Fertil Steril.

[18] Prisant N, Tubiana R, Lefebvre G, Lebray P, Marcelin AG, Thibault V (2010). HIV-1 or hepatitis C chronic infection in serodiscordant infertile couples has no impact on infertility treatment outcome. Fertil Steril.

[19] Shenfield F, Pennings G, Cohen J, Devroey P, Tarlatzis B, Sureau C (2004). Taskforce 8: ethics of medically assisted fertility treatment for HIV positive men and women. Hum Reprod.

[20] World Health Organization (1999). World Health Organization laboratory manual for the examination of human semen and sperm-cervical mucus interaction.

[21] Palermo GD, Cohen J, Alikani M, Adler A, Rosenwaks Z (1995). Development and implementation of intracytoplasmic sperm injection (ICSI). Reprod Fertil Dev.

[22] Zegers-Hochschild F, Adamson GD, de Mouzon J, Ishihara O, Mansour R, Nygren K (2009). The international committee for monitoring assisted reproductive technology (ICMART) and the World Health Organization (WHO) revised glossary on ART terminology, 2009. Hum Reprod.

[23] Vernazza P, Hirshel B, Bernasconi E, Flepp M (2008). Les personnes seropositives ne souffrant d’aucune autre MST et suivant un traitement antiretroviral efficace ne transmettent pas le HIV par voie sexuelle. Bull Med Suisses.

[24] Gilling-Smith C (2000). HIV prevention.Assisted reproduction in HIVdiscordant couples. AIDS Read.

[25] Garrido N, Meseguer M (2006). Use of washed sperm for assisted reproduction in HIV-positive males without checking viral absence.A risky business. Hum Reprod.

[26] Savasi V, Ferrazzi E, Lanzani C, Oneta M, Parrilla B, Persico T (2007). Safety of sperm washing and ART outcome in 741 HIV-1-serodiscordant couples. Hum Reprod.

[27] Neumayr G, Propst A, Schwaighofer H, Judmaier G, Vogel W (1999). Lack of evidence for the heterosexual transmission of hepatitis C. QJM.

[28] Marincovich B, Castilla J, del Romero J, Garcia S, Hernando V, Raposo M (2003). Absence of hepatitis C virus transmission in a prospective cohort of heterosexual serodiscordant couples. Sex Transm Infect.

[29] Wasley A, Grytdal S, Gallagher K (2008). Surveillance for acute viral hepatitis--United States, 2006. MMWR Surveill Summ.

[30] Anderson JR (2005). A guide to the clinical care of women with HIV/ AIDS.

[31] Mast EE, Margolis HS, Fiore AE, Brink EW, Goldstein ST, Wang SA (2005). A comprehensive immunization strategy to eliminate transmission of hepatitis B virus infection in the United States: recommendations of the Advisory Committee on Immunization Practices (ACIP) part 1: immunization of infants, children, and adolescents. MMWR Recomm Rep.

[32] Pasquier C, Daudin M, Righi L, Berges L, Thauvin L, Berrebi A (2000). Sperm washing and virus nucleic acid detection to reduce HIV and hepatitis C virus transmission in serodiscordant couples wishing to have children. AIDS.

[33] Piomboni P, Baccetti B (2000). Spermatozoon as a vehicle for HIV- 1 and other viruses: a review. Mol Reprod Dev.

[34] Gordon JW (2002). Micromanipulation of gametes and embryos may be a risk for human germ-line gene transfer. Fertil Steril.

[35] Anderson DJ, Politch JA (2003). Providing fertility care to HIV-1 serodiscordant couples: a biologist’s point of view. Am J Bioeth.

[36] Ohl J, Partisani M, Wittemer C, Schmitt MP, Cranz C, Stoll- Keller F (2003). Assisted reproduction techniques for HIV serodiscordant couples: 18 months of experience. Hum Reprod.

[37] Kato S, Hanabusa H, Kaneko S, Takakuwa K, Suzuki M, Kuji N (2006). Complete removal of HIV-1 RNA and proviral DNA from semen by the swim-up method: assisted reproduction technique using spermatozoa free from HIV-1. AIDS.

[38] Chrystie IL, Mullen JE, Braude PR, Rowell P, Williams E, Elkington N (1998). Assisted conception in HIV discordant couples: evaluation of semen processing techniques in reducing HIV viral load. J Reprod Immunol.

[39] Semprini AE, Persico T, Thiers V, Oneta M, Tuveri R, Serafini P (1998). Absence of hepatitis C virus and detection of hepatitis G virus/GB virus C RNA sequences in the semen of infected men. J Infect Dis.

[40] Debono E, Halfon P, Bourliere M, Gerolami-Santandrea V, Gastaldi M, Castellani P (2000). Absence of hepatitis C genome in semen of infected men by polymerase chain reaction, branched DNA and in situ hybridization. Liver.

[41] Tang Z, Yang D, Hao L, Huang Y, Wang S (1996). Detection and significance of HCV RNA in saliva, seminal fluid and vaginal discharge in patients with hepatitis C. J Tongji Med Univ.

[42] Levy R, Tardy JC, Bourlet T, Cordonier H, Mion F, Lornage J (2000). Transmission risk of hepatitis C virus in assisted reproductive techniques. Hum Reprod.

[43] Bourlet T, Levy R, Laporte S, Blachier S, Bocket L, Cassuto G (2003). Multicenter quality control for the detection of hepatitis C virus RNA in seminal plasma specimens. J Clin Microbiol.

[44] Pasquier C, Souyris C, Moinard N, Bujan L, Izopet J (2006). Validation of an automated real-time PCR protocol for detection and quantitation of HIV and HCV genomes in semen. J Virol Methods.

[45] Manigart Y, Rozenberg S, Barlow P, Gerard M, Bertrand E, Delvigne A (2006). ART outcome in HIV-infected patients. Hum Reprod.

[46] Nicopoullos JD, Almeida P, Vourliotis M, Goulding R, Gilling- Smith C (2010). A decade of the sperm-washing programme: where are we now. Hum Fertil (Camb).

[47] Dulioust E, Du AL, Costagliola D, Guibert J, Kunstmann JM, Heard I (2002). Semen alterations in HIV-1 infected men. Hum Reprod.

[48] Nicopoullos JD, Almeida PA, Ramsay JW, Gilling-Smith C (2004). The effect of human immunodeficiency virus on sperm parameters and the outcome of intrauterine insemination following sperm washing. Hum Reprod.

[49] Aflatoonian A, Oskouian H, Ahmadi S, Oskouian L (2010). Can fresh embryo transfers be replaced by cryopreserved-thawed embryo transfers in assisted reproductive cycles?. J Assist Reprod Genet.

[50] Shapiro BS, Daneshmand ST, Garner FC, Aguirre M, Ross R (2008). Contrasting patterns in in vitro fertilization pregnancy rates among fresh autologous, fresh oocyte donor, and cryopreserved cycles with the use of day 5 or day 6 blastocysts may reflect differences in embryo-endometrium synchrony. Fertil Steril.

[51] Melo MA, Meseguer M, Bellver J, Remohi J, Pellicer A, Garrido N (2008). Human immunodeficiency type-1 virus (HIV-1) infection in serodiscordant couples (SDCs) does not have an impact on embryo quality or intracytoplasmic sperm injection (ICSI) outcome. Fertil Steril.

[52] Schieve LA, Meikle SF, Ferre C, Peterson HB, Jeng G, Wilcox LS (2002). Low and very low birth weight in infants conceived with use of assisted reproductive technology. N Engl J Med.

[53] Centers for Disease Control and Prevention, American Society
for Reproductive Medicine, Society for Assisted Reproductive
Technology (2010). 2008 Assisted Reproductive Technology
Success Rates: National Summary and Fertility Clinic Reports.

